# Factors Associated With COVID-19 Vaccine Receipt by Health Care Personnel at a Major Academic Hospital During the First Months of Vaccine Availability

**DOI:** 10.1001/jamanetworkopen.2021.36582

**Published:** 2021-12-01

**Authors:** Judith Green-McKenzie, Frances S. Shofer, Florence Momplaisir, Barbara J. Kuter, Gregory Kruse, Usama Bialal, Maryam Behta, Judith O’Donnell, Nida Al-Ramahi, Nishaminy Kasbekar, Patricia Sullivan, Philip Okala, Patrick J. Brennan

**Affiliations:** 1 Leonard Davis Institute, Division of Occupational Medicine, Department of Emergency Medicine, University of Pennsylvania Perelman School of Medicine, Philadelphia; 2 Epidemiology and Biostatistics Research, Department of Emergency Medicine, University of Pennsylvania Perelman School of Medicine, Philadelphia; 3 Leonard Davis Institute, Department of Infectious Disease, University of Pennsylvania Perelman School of Medicine, Philadelphia; 4 Vaccine Education Center, Children’s Hospital of Philadelphia, Philadelphia, Pennsylvania; 5 University of Pennsylvania Perelman School of Medicine, Philadelphia; 6 Urban Health Collaborative and Department of Epidemiology and Biostatistics, Drexel Dornsife School of Public Health, Philadelphia, Pennsylvania; 7 University of Pennsylvania Health System, Philadelphia; 8 Infection Control, Penn Presbyterian Medical Center, Division of Infectious Diseases, Clinical Medicine, University of Pennsylvania Perelman School of Medicine, Philadelphia; 9 Office of the Chief Medical Officer and Chief Quality Officer, University of Pennsylvania Health System, Philadelphia; 10 Penn Presbyterian Medical Center, Philadelphia, Pennsylvania

## Abstract

**Importance:**

Several COVID-19 vaccines have been authorized in the US, yet preliminary evidence suggests high levels of vaccine hesitancy and wide racial, ethnic, and socioeconomic disparities in uptake.

**Objective:**

To assess COVID-19 vaccine acceptance among health care personnel (HCP) during the first 4 months of availability in a large academic hospital, compare acceptance with previously measured vaccine hesitancy, and describe racial, ethnic, and socioeconomic disparities in vaccine uptake.

**Design, Setting, and Participants:**

This cross-sectional study included 12 610 HCP who were offered COVID-19 vaccination at a major academic hospital in Philadelphia between December 16, 2020, and April 16, 2021.

**Exposures:**

For each HCP, data were collected on occupational category, age, sex, race and ethnicity (Asian or Pacific Islander, Black or African American [Black], Hispanic, White, and multiracial), and social vulnerability index (SVI) at the zip code of residence.

**Main Outcomes and Measures:**

Vaccine uptake by HCP at the employee vaccination clinic.

**Results:**

The study population included 4173 men (34.8%) and 7814 women (65.2%) (623 without data). A total of 1480 were Asian or Pacific Islander (12.4%); 2563 (21.6%), Black; 452 (3.8%), Hispanic; 7086 (59.6%), White; and 192 (1.6%), multiracial; 717 had no data for race and ethnicity. The mean (SD) age was 40.9 (12.4) years, and 9573 (76.0%) received at least 1 vaccine dose during the first 4 months of vaccine availability. Adjusted for age, sex, job position, and SVI, Black (relative risk [RR], 0.69; 95% CI, 0.66-0.72) and multiracial (RR, 0.80; 95% CI, 0.73-0.89) HCP were less likely to receive vaccine compared with White HCP. When stratified by job position, Black nurses (n = 189; 62.8%), Black HCP with some patient contact (n = 466; 49.9%), and Black HCP with no patient contact (n = 636; 56.3%) all had lower vaccine uptake compared with their White and Asian or Pacific Islander counterparts. Similarly, multiracial HCP with some (n = 26; 52.0%) or no (n = 48; 58.5%) patient contact had lower vaccine uptake. In contrast, Black physicians were just as likely to receive the vaccine as physicians of other racial and ethnic groups.

**Conclusions and Relevance:**

In this cross-sectional study, more than two-thirds of HCP at a large academic hospital in Philadelphia received a COVID-19 vaccine within 4 months of vaccine availability. Although racial, ethnic, and socioeconomic disparities were seen in vaccine uptake, no such disparities were found among physicians. Better understanding of factors driving these disparities may help improve uptake.

## Key Points

**Question:**

What was the COVID-19 vaccine uptake among health care personnel (HCP) shortly after availability, and what factors were associated with uptake?

**Findings:**

In this cross-sectional study of 12 610 HCP at a major US academic hospital, two-thirds received a first dose within the first 4 months; 98% of those received 2 doses. Adjusted for age, sex, job position, and area-level social vulnerability, Black or African American and multiracial HCP were less likely to receive the vaccine compared with White HCP, with narrower disparities observed for nurses and no disparities found among physicians.

**Meaning:**

These findings indicate the presence of racial and ethnic disparities in HCP vaccine uptake, except among physicians.

## Teaser

This cross-sectional study examines COVID-19 vaccine uptake among health care personnel and assesses factors associated with uptake, including racial, ethnic, and socioeconomic disparities.

## Introduction

The first vaccine against SARS-CoV-2 virus was granted emergency use authorization in the US and became available for use on December 11, 2020.^1^ This Pfizer-BioNTech COVID-19 vaccine was deployed to the US population through local, regional, territorial, and state departments of health in a tiered manner using a priority group system, based on recommendations of the federal Advisory Committee on Immunization Practices. Issues considered for the prioritization included the risk of exposure and likelihood of severe disease. The Centers for Disease and Control and Prevention recommended vaccines be distributed using a phased system with 4 priority groups: phases 1a, 1b, 1c, and 2. Health care personnel (HCP) were in phase 1a.

Health care personnel at a large teaching hospital in Philadelphia, Pennsylvania were offered the Pfizer-BioNTech vaccine as soon it became available.^2,3^ Health care personnel constitute a unique group, because the personal risk of infection must be considered alongside the possibility of transmitting an infectious agent, such as the SARS-CoV-2 virus, to patients. Coexistence of HCP and patients in an enclosed setting lends itself to rapid dissemination of the SARS-CoV-2 virus.^4,5,6^

Nationwide surveys through 2020 suggested that COVID-19 vaccine hesitancy was significant, changing over time from July to December 2020.^7^ A survey administered to HCP at this hospital, simultaneously with another teaching hospital in Philadelphia in November 2020, sought to determine the degree to which vaccine hesitancy might exist among HCP.^8^ Vaccination intent was assessed, and it was found that 74.1% of Asian or Pacific Islander HCP, 29.7% of Black HCP, 54.4% of Hispanic HCP, 69.5% of White HCP, and 58.8% of multiracial HCP intended to be vaccinated. The main reported concerns about vaccine receipt were side effects, the vaccine being new, and not knowing enough about the vaccine.^8^ Strategies to reduce hesitancy were discussed at the COVID-19 Vaccine Advisory Committee of the Hospital, chaired by the Chief Medical Officer, and implemented based on these survey results. There are limited data on strategies shown to improve vaccination rates among HCP who are hesitant to receive the COVID-19 vaccine. A comprehensive framework to understand barriers and facilitators associated with vaccine hesitancy among HCP is important.

The purpose of this research was to (1) assess vaccine uptake in the first few months after vaccine rollout among HCP at this hospital; (2) assess disparities in early adoption of vaccination by race and ethnicity, including examining the association of socioeconomic status (SES) with vaccine acceptance using geographic location of residence as a proxy; (3) determine any association between the prevaccine survey results describing HCP vaccine hesitancy and vaccine acceptance at the same hospital; and (4) discuss health-system strategies to decrease vaccine hesitancy and increase vaccine uptake.

## Methods

This cross-sectional study examined HCP vaccine uptake at the hospital’s employee vaccination clinic during the early months of vaccine availability and is reported using the Strengthening the Reporting of Observational Studies in Epidemiology (STROBE) reporting guideline for cross-sectional studies. This research was deemed exempt by the University of Pennsylvania Perelman School of Medicine Institutional Review Board, with a waiver of informed consent granted for the use of deidentified data.

### Study Population and Period

The study population consisted of HCP, defined as all paid employees, at a major academic hospital. All hospital employees were eligible for the Pfizer-BioNTech mRNA SARS-CoV-2 vaccine, the only COVID-19 vaccine offered at this hospital once it became available under the emergency use authorization.^1^ The study period was the first 4 months of vaccine availability at the hospital, from Wednesday, December 16, 2020 (the day the first doses were administered), to Friday, April 16, 2021.

### Outcomes of Interest and Exposure Variables

The primary outcomes of interest were uptake of the first and second vaccine doses during the study period. Deidentified data on the number of vaccine doses administered to each HCP was obtained from individual electronic medical records at the hospital.

The demographic variables obtained on each employee included age, sex, race and ethnicity, zip code of residence, and job category. Employees self-identified their sex as male or female, and their race as Asian or Pacific Islander, Black or African American–non-Hispanic (Black), Hispanic or Latino (Hispanic), White–non-Hispanic, multiracial, or other race (included Alaskan Native or American Indian and those identifying as other race with no specification). Four job categories were created: (1) physicians, (2) nurses (advanced practice, licensed practical, and registered nurses), (3) HCP with some patient contact (eg, technicians, therapists, nursing aides, phlebotomists), and (4) HCP with no designated patient contact (eg, finance, coders, information technology, environmental and food services). Job categories that could not be clearly categorized were not included for the analysis using job category.

The Social Vulnerability Index (SVI), a composite index reflecting a community’s ability to prevent human suffering and financial loss in the event of disaster, including disease outbreak,^9^ was used to measure each employee’s social conditions in their neighborhood of residence. The SVI includes 4 domains: SES, household composition and disability, minority status and language, and housing type and transportation. Tertiles ranging from high, signifying highest social vulnerability, to low, signifying lowest social vulnerability, were created.

### Prioritization Scheme

Health care personnel were in the first group recommended for COVID-19 vaccination by the Advisory Committee on Immunization Practices^2^ and eligible as soon as vaccine was available. Further prioritization was necessary at our hospital, given the initial limited vaccine supply. The hospital’s COVID-19 Vaccine Advisory Group, comprised of experts from various specialties, applied ethical principles to make prioritization determinations. Prioritization determination, based on the Centers for Disease Control and Prevention’s guidelines, included likelihood of exposure to aerosols during patient care, frequency of exposure to the oropharynx and respiratory tract mucosa, duration of time spent in direct patient care delivery, and the degree of contact with certain highly vulnerable patient populations. The timing of HCP vaccination was carried out according to their level of risk during patient contact. Using this schema, HCP were offered the vaccine at 5 points in time: December 16, 20, and 26, 2020; and January 4 and 7, 2021 (eTable in the Supplement). All HCP had the opportunity to schedule vaccination within 4 weeks of vaccine arrival. Capacity was built to allow administration of hundreds of vaccine doses daily.

### Vaccine Availability Messaging and Community COVID-19 Vaccine Clinics

Six weeks before vaccine arrival, weekly messaging was distributed to employees describing the vaccine rollout process, providing facts about vaccine safety and efficacy, and informing employees to be ready for the vaccine when offered. Messaging was delivered through news tickers; systemwide emails; town halls, one of which focused on addressing concerns specifically within the Black and Hispanic communities; and one-on-one huddles, geared toward HCP with some or no patient contact, with physician volunteers who were knowledgeable about the COVID-19 vaccine.^10^

Weekly community vaccination clinics, located in predominantly African American neighborhoods proximal to the hospital, were launched,^11^ beginning 13 weeks after vaccine arrival and continuing throughout the study period. Black employees living in these communities could potentially interact with vaccinated community members and see them as role models for vaccine acceptance.

### Statistical Analysis

Summary statistics are presented as frequencies and percentages for all demographic parameters. The χ^2^ test was used to compare HCP who received the vaccine vs those who did not by sex, age group, race and ethnicity, and SVI. Within the 4 job categories (physician, nursing, HCP with some and no direct patient contact), the same sets of comparisons were performed as for the entire group. To examine differences over time in vaccination rates, cumulative vaccine rates were plotted over week of vaccination and stratified by race and ethnicity. Similar plots were created by HCP job category. To assess whether race was independently associated with vaccine uptake while adjusting for age, sex, job category, and SVI, relative risk (RR) was estimated using a generalized linear model with a log link, Gaussian error, and robust estimates of the standard errors of the model coefficient.^12^ Variable selection was defined a priori. These analyses were performed using STATA, version 15 (StataCorp LLC). Data for these analyses are presented as RRs with 95% CIs. To examine difference in actual uptake of vaccine vs plans to get vaccinated in the earlier survey, a χ^2^ test was performed. All tests were 2-sided, and *P* < .05 was considered statistically significant. Unless otherwise noted, all analyses were performed using SAS statistical software, version 9.4 (SAS Institute, Inc).

## Results

### Study Population Demographic Characteristics

Of the 12 610 HCP, 4173 (34.8%) were men and 7814 (65.2%) were women (623 without data). A total of 1480 (12.4%) were Asian or Pacific Islander; 2563 (21.6%), Black; 452 (3.8%), Hispanic; 7086 (59.6%), White; 192 (1.6%), multiracial; and 120 (1.0%), other race; 717 had no data for race and ethnicity. The mean (SD) age was 40.9 (12.4) years (Table 1). During the first 4 months of vaccine availability, 9573 HCP (76.0%) received at least 1 dose of the vaccine, with 9207 of 9373 who were eligible during the study period (98.0%) receiving a second dose. Black (n = 1440; 56.2%) and multiracial (n = 123; 64.1%) HCP were less likely to be vaccinated compared with Asian or Pacific Islander HCP (n = 1306; 88.3%), Hispanic HCP (n = 363; 80.3%), White HCP (n = 6157; 86.9%), and HCP identifying as other race (n = 109; 90.8%). Younger HCPs (18-24 years old) were less likely to be vaccinated than HCP aged 25 years or older (287 [61.6%] vs 3452 [76.5%]). Employees living in areas with a high SVI were less likely to be vaccinated compared with those living in areas with a low SVI (3080 [69.5%], 2671 [82.0%], and 3168 [84.6%] for high, medium, and low SVI, respectively).

**Table 1.** Vaccine Acceptance Rates Among HCP Offered COVID-19 Vaccine by Demographic Characteristics^a^

Characteristic	Total^b^	No. (%)
		Received first vaccine dose
All HCP	12 610	9573 (76.0)
Sex		
Female	7814	6137 (78.5)
Male	4173	3436 (82.3)
Age, y		
Mean (SD)	40.9 (12.4)	NA
18-24	466	287 (61.6)
25-34	4516	3453 (76.5)
35-44	3222	2411 (74.9)
45-54	2163	1636 (75.6)
55-64	1737	1387 (80.0)
≥65	506	399 (78.9)
Race and ethnicity		
Asian or Pacific Islander	1480	1306 (88.2)
Black or African American (non-Hispanic)	2563	1440 (56.2)
Hispanic or Latino	452	363 (80.3)
White (non-Hispanic)	7086	6157 (86.9)
Multiracial	192	123 (64.1)
Other race^c^	120	109 (90.8)
Social Vulnerability Index^d^		
Low	3744	3168 (84.6)
Medium	3257	2671 (82.0)
High	4434	3080 (69.5)
Job position		
Physicians	2788	2415 (86.6)
Nurses	3132	2707 (86.3)
Some patient contact^e^	2502	1460 (58.4)
No patient contact^f^	3931	2893 (73.6)

Abbreviations: HCP, health care personnel; NA, not applicable.

^a^ All differences in vaccine uptake determined by χ^2^ tests; *P* < .001 for all.

^b^ Ns may not total to full sample due to missing values (sex: n = 623, 4.9%; race and ethnicity: n = 717, 5.7%; SVI: n = 1175, 9.3%; job position: n = 257, 2%).

^c^ Includes Alaskan Native, American Indian, and those identifying as other.

^d^ Approximation based on zip code of residence of employee.

^e^ Includes environmental and food services, transporters, security, nursing aides, social workers, dietitians, physical therapists, radiology technicians, phlebotomists, and others.

^f^ Includes administrative, financial, clerical, management, clinical laboratory personnel, information technology, maintenance, and pharmacy.

Health care personnel with direct patient care were more likely to be vaccinated (including 2415 physicians [86.6%] and 2707 nurses [86.3%]) compared with HCP with some patient contact (1460 [58.4%]) and no patient contact (2893 [73.6%] (Table 1). Although Black physicians were as likely as all other physicians to be vaccinated (Table 2), Black nurses were less likely to be vaccinated (n = 189; 62.8%) compared with all others. Health care personnel with some and no patient contact showed similar racial and ethnic disparities: Black HCP with some patient contact (n = 466; 49.9%) and Black HCP with no patient contact (n = 636; 56.3%) all had lower vaccine uptake compared with their White and Asian or Pacific Islander counterparts. Health care personnel with some or no patient contact living in areas with a high SVI (584 of 1064 [54.9%] and 1112 of 1707 [65.1%], respectively) were less likely to be vaccinated compared with those living in areas with a medium SVI (306 of 446 [68.6%] and 767 of 972 [78.9%], respectively) or low SVI (373 of 478 [78.0%] and 946 of 1097 [86.2%], respectively) (Table 2).

**Table 2.** Comparison of Health Care Personnel by Demographic Variables Stratified by Job Category^a^

Variable	Physicians (n = 2788)			Nurses (n = 3132)			Some patient contact (n = 2502)			No patient contact (n = 3931)		
	Total	Received first vaccine dose, No. (%)	*P* value^b^	Total	Received first vaccine dose, No. (%)	*P* value^b^	Total	Received first vaccine dose, No. (%)	*P* value^b^	Total	Received first vaccine dose, No.(%)	*P* value^b^
Sex												
Female	1117	1029 (92.1)	.36	2731	2364 (86.6)	>.99	1534	1025 (66.8)	.73	2278	1653 (72.6)	<.001
Male	1489	1386 (93.1)		390	338 (86.7)		659	435 (66)		1568	1240 (79.1)	
Unknown	182	0		11	0		309	0		85	0	
Age, y												
18-34	1049	952 (90.8)	<.001	1735	1503 (86.6)	.76	867	417 (48.1)	<.001	1192	815 (68.4)	<.001
35-44	783	669 (85.4)		773	659 (85.3)		679	394 (58.0)		939	667 (71)	
45-54	439	365 (83.1)		400	344 (86)		492	318 (64.6)		798	597 (74.8)	
≥55	517	429 (83.0)		224	196 (87.5)		464	331 (71.3)		1002	814 (81.2)	
Race and ethnicity												
Asian or Pacific Islander	588	540 (91.8)	.46	314	279 (88.9)	<.001	121	101 (83.5)	<.001	438	378 (86.3)	<.001
Black or African American (non-Hispanic)	139	134 (96.4)		301	189 (62.8)		934	466 (49.9)		1130	636 (56.3)	
Hispanic or Latino	101	94 (93.1)		93	83 (89.3)		79	58 (73.4)		167	124 (74.3)	
White (non-Hispanic)	1631	1507 (92.4)		2366	2111 (89.2)		966	776 (80.3)		2003	1693 (84.5)	
Multiracial	10	10 (100)		40	35 (87.5)		52	26 (52.0)		82	48 (58.5)	
Other race^c^	85	80 (94.1)		3	3 (100)		17	15 (88.2)		13	10 (76.9)	
Unknown	234	50 (21.4)		15	2 (13.3)		333	18 (5.4)		98	4 (4.1)	
SVI^d^												
Low	768	655 (85.3)	.21	1334	1161 (87.0)	.35	478	373 (78.0)	<.001	1097	946 (86.2)	<.001
Medium	870	771 (88.6)		911	793 (87.1)		446	306 (68.6)		972	767 (78.9)	
High	717	627 (87.5)		856	728 (85.1)		1064	584 (54.9)		1707	1112 (65.1)	
Unknown	433	362 (83.6)		31	20 (64.5)		514	197 (38.3)		155	68 (43.9)	

Abbreviations: HCP, health care personnel; SVI, Social Vulnerability Index.

^a^ Job category assigned to all but 2% (257) of HCP.

^b^ Statistical comparisons used χ^2^ tests. Unknown values were not included in statistical testing.

^c^ Includes Alaskan Native, American Indian, and those identifying as other.

^d^ Social Vulnerability Index: Approximation based on zip code of residence of employee.

### Vaccine Uptake During the Study Period

There was an absolute increase in actual HCP vaccine uptake compared with the reported intention to be vaccinated reported in an earlier survey.^8^ The largest increase was seen in Black HCP (27%), followed by Hispanic (26%), multiracial (16%), White (17%), and Asian or Pacific Islander (14%) HCP. There was an 18% increase in acceptance by women, with no change for men.

**Figure 1.**
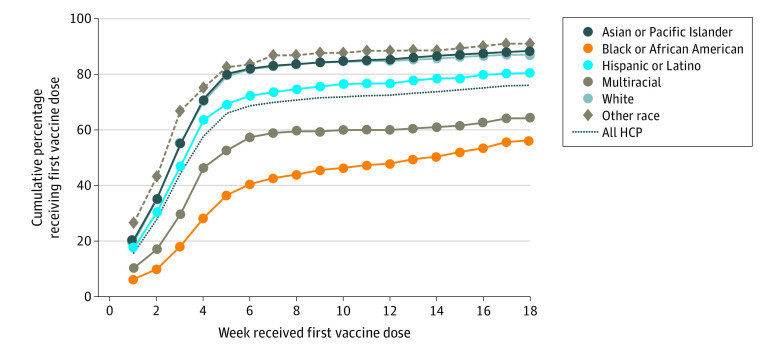
Weekly Cumulative Percent of Health Care Personnel Receiving the First Vaccine Dose by Race and Ethnicity Other race Includes Alaskan Native, American Indian, and those identifying as other.

**Figure 2.**
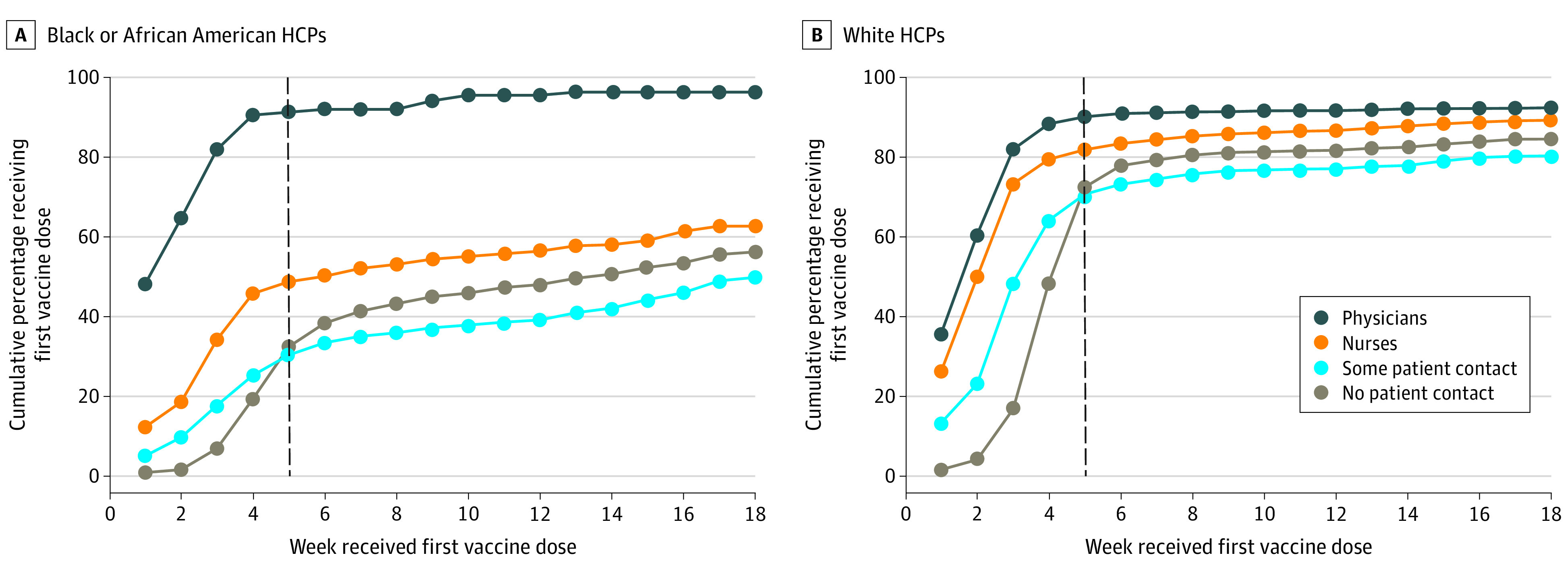
Weekly Cumulative Percent of Health Care Personnel (HCP) Receiving the First Vaccine Dose by Race and Ethnicity Stratified by Job Category The dashed line indicates when 70% of HCP were vaccinated. For White individuals, all HCP regardless of job category achieved 70% by 5 weeks, whereas among Black and African American HCPs, only physicians reached the 70% threshold.

Among the 9573 HCP who received the COVID-19 vaccine within the first 4 months of availability, the number vaccinated rose exponentially during the first 5 weeks for all groups, reaching an overall 50% vaccination rate by week 4. (Figure 1). Among HCP receiving the vaccine, 366 of 1440 Black HCP (25.4%) received their vaccine (weeks 5-7) compared with a combined 1148 of 8058 in the other racial and ethnic groups (14.2%). After the launch of the community clinic (week 13), 213 of 1440 Black HCP (15.0%) compared with 234 of 8058 HCP in other groups (3.0%) received the vaccine (weeks 13-18) (Figure 2). Black physicians reached a 91% vaccination rate by week 5 (127 of 139 physicians), compared with 49% of nurses (147 of 301), 32% of HCP with no patient contact (367 of 1130), and 30% of HCP with some patient contact (284 of 934).

### Adjusted Logistic Regression Results

When adjusted for sex, age, occupational category, and SVI, Black HCP were 31% less likely (RR, 0.69; 95% CI, 0.66-0.72) and multiracial HCP were 20% less likely (RR, 0.80; 95% CI, 0.73-0.89) to be vaccinated during the first 4 months compared with White HCP. Personnel living in areas with a high SVI had a 3% lower uptake vs those in areas with a low SVI after adjusting for all other demographic variables (RR, 0.97; 95% CI, 0.95-0.99) (Table 3).

**Table 3.** Relative Risk Regression Models, Modeled on 11 063 Health Care Personnel Who Received the First Vaccine Dose

Variable	Unadjusted		Adjusted	
	Risk ratio (95% CI)	*P* value	Risk ratio (95% CI)	*P* value
Race and ethnicity				
Asian or Pacific Islander	1.01 (0.99-1.04)	.20	1.01 (0.99-1.04)	.22
Black or African American (non-Hispanic)	0.63 (0.61-0.66)	<.001	0.69 (0.66-0.72)	<.001
Hispanic or Latino	0.93 (0.89-0.98)	.003	0.97 (0.93-1.01)	.16
White (non-Hispanic)	1 [Reference]	NA	1 [Reference]	NA
Multiracial	0.74 (0.67-0.83)	<.001	0.80 (0.73-0.89)	<.001
Other	1.02 (0.95-1.11)	.56	1.01 (0.99-1.04)	.62
Sex				
Male	NA		1.00 (0.98-1.02)	.81
Female	NA		1 [Reference]	
Age, y				
18-24	NA		1 [Reference]	NA
35-44	NA		1.00 (0.98-1.03)	.69
45-54	NA		1.03 (1.01-1.06)	.009
55-64	NA		1.10 (1.07-1.13)	<.001
≥65	NA		1.13 (1.09-1.17)	<.001
Job or position				
No patient contact	NA		1 [Reference]	NA
Physician	NA		1.12 (1.09-1.14)	<.001
Nurse	NA		1.09 (1.07-1.12)	<.001
Some patient contact	NA		0.91 (0.88-0.94)	<.001
SVI^a^				
Low	NA		1 [Reference]	NA
Medium	NA		1.00 (0.98-1.02)	.73
High	NA		0.97 (0.95-0.99)	.004

Abbreviation: SVI, Social Vulnerability Index.

^a^ Approximation based on zip code of residence of employee.

## Discussion

In this cross-sectional study of HCP at a major US academic hospital, vaccine uptake was not uniform across all races and ethnicities. Consistent with some national data,^7^ Black HCP had substantially lower uptake than other groups, followed by multiracial and Hispanic HCP. Asian or Pacific Islander and White HCP had the highest uptake. The number of HCP accepting the COVID-19 vaccine rose exponentially for all employees during the first 5 weeks. All HCP had been invited to be vaccinated by week 4through multiple means of messaging. Despite vaccine availability, vaccination capacity, and on-the-job access, approximately one-quarter of HCP remained unvaccinated after 4 months. Indeed, by the fourth month, HCP demand dropped for all groups.

Our study confirmed prevaccination survey findings that more older HCP planned to be vaccinated than younger HCP.^8^ This disparity held for all groups except physicians. Younger physicians were more likely to be vaccinated, possibly because house staff, who are more likely to be engaged in direct patient care most of the time, are younger. Physicians no longer in training have varied duties with potentially reduced patient care roles and may feel less urgency to be vaccinated early.

Hospital outreach and educational efforts may have helped some HCP overcome their hesitancy as vaccine coverage rates exceeded reported intention to be vaccinated,^8^ with the greatest increase seen for Black and Hispanic HCP. Another potential reason for this association could be secular trends in vaccine acceptance following the publication of clinical trial results. Steep increases in vaccination rates were observed for nonphysician Black HCP during weeks 5 through 7 (when town halls and huddles were implemented) and weeks 15 through 18 (when community clinics were launched^13^). The temporal association of these focused interventions with an increase in COVID-19 vaccine acceptance suggests added value, but the association is certainly not causal (Figure 1). Having questions answered in small groups, hearing from trusted messengers at the town hall, and having the peer influence of seeing family or friends in the community vaccinated may have lessened concerns about the vaccine.^8^

Health care personnel with limited patient contact living in areas with a high SVI were less likely to be vaccinated. Although some reasons reported elsewhere for not receiving a COVID-19 vaccine include lack of access and economic barriers,^14^ the vaccine was given at no cost to this HCP population and was readily accessible at work. Thus, this finding is concerning because it suggests other potential barriers, such as a lack of medical knowledge on vaccine benefits, persisting concerns regarding safety and efficacy,^15,16^ or other unidentified factors.

Interestingly, all physicians were as likely to accept the vaccine regardless of race or ethnicity, with a greater than 90% uptake rate within our 4-month study period. Nurses of all races and ethnicities were as likely to take the vaccine except for Black nurses, who were less likely to do so. There was less consistency across job positions for HCP with some or no direct patient contact (Figure 2).

The finding that there were no racial or ethnic disparities in COVID-19 vaccine acceptance among physicians but wide racial and ethnic disparities among nurses and HCP with some or no patient contact supports the idea that these disparities are not simply a function of race and ethnicity and that their causes are more complex.^17,18,19^ That all physicians were equally likely to receive a COVID-19 vaccine regardless of race or ethnicity may be due their medical education,^20^ knowledge of science, and belief in the value of vaccines as a key disease prevention tool. This possibility would not be surprising, as it has been shown that vaccine acceptance increased after the publication of trials demonstrating high vaccine efficacy.^16^ Disparities persisted regarding SVI, which was associated with lower uptake after adjusting for race suggesting that vaccine acceptance is associated with occupational and neighborhood conditions as well. Indeed, SVI made a significant difference only with HCP who had some or no patient contact.

Of note, job title (occupational class) is one of the main components of individual-level SES, whereas area-level SVI is a marker (albeit an imperfect one) for SES, given strong economic segregation patterns.^14^ Therefore, both the physician and nurse groups are more homogeneous categories in terms of SES (and, therefore, SVI), as within each group all individuals belong to the same occupational class. However, the group of other HCP with some or no patient contact represents a heterogeneous group in terms of SES (eg, physical, occupational, and behavioral therapists vs food service workers), leading to wider variability in SVI. The finding that SVI was associated with vaccination uptake among these more heterogeneous groups could be explained by this wider variability in SVI, whereas SVI not being associated with physician or nurse vaccination is likely because these groups have more homogeneous SES. However, we did not have data on individual-level SES, which may help clarify within-group differences. Our prevaccine survey, which identified respondents’ 3 top reasons for not wanting to get vaccinated (possible side effects, the vaccine being too new, and not knowing enough about the vaccine),^8^ may have been useful in helping hospital leadership better address HCP concerns regarding receipt of a new COVID-19 vaccine. Several initiatives instituted to address these concerns may have, at least partially, helped to alleviate HCP concerns and improve vaccination rates.

Although we were able to provide vaccines to 76% of HCP during the first 4 months of our vaccination program, work remains to be done, as 24% of the employee population is still not vaccinated despite capacity and availability. Although this percentage includes HCP who are on leave of absence, ill, or had reasons not to be vaccinated at the time but may be eligible in the future, it may also include the hesitant.

### Strengths and Limitations

Study results are strengthened by the large sample size, vaccine accessibility to all HCP, and the ability to compare vaccination intent and uptake on a population basis closely in time. Survey results helped to inform vaccination strategy and have not been previously described. A comprehensive measure of social factors at the neighborhood level (SVI), shown to be highly predictive of COVID-19 outcomes, was used.^9^

The study was limited in that prevaccination survey results could not be linked to vaccine uptake at an individual level owing to privacy issues. Only group data were available. Nonetheless, the survey queried a large enough sample of our hospital population to be representative. The increase in vaccine acceptance after the survey is merely a descriptive result, not suggestive of any causal relationship, as are any associations seen with interventions made. Our results originated from an urban academic hospital and therefore may not be generalizable to all health care institutions. As described above, area-level SVI measures are imperfect and may result in misclassification.^9^ It is possible that HCP could have been vaccinated elsewhere, resulting in incomplete ascertainment. However, we expect that number to be small given the easy access to vaccines at work.

## Conclusions

In summary, this cross-sectional study found that more than three-quarters of HCP received a COVID-19 vaccine within 4 months of availability, more than half within the first 4 weeks. Educational outreach may have increased vaccine uptake. Racial and ethnic disparity, evident among nonphysicians with significantly lower uptake among Black HCP, was not seen among physicians. Health care personnel with some or no patient contact and those living in areas with a high SVI had lower vaccine uptake. With a mandatory vaccine policy now a reality at this hospital, the hope is that unvaccinated HCP will decide to be vaccinated and hospital leadership can adequately address reasons for hesitancy.

## Notes

Supplement. **eTable.** Employee Prioritization Sequence Grid for Time to Receive COVID-19 Vaccine
