# Error in Author’s Name

**DOI:** 10.1001/jamanetworkopen.2021.47879

**Published:** 2022-01-24

In the Original Investigation titled “Factors Associated With COVID-19 Vaccine Receipt by Health Care Personnel at a Major Academic Hospital During the First Months of Vaccine Availability,”^1^ published December 1, 2021, author Usama Bilal’s surname was incorrectly spelled as Bialal in the byline and affiliations. This article has been corrected.^1^
