# Supplementary material for: Factors Associated With COVID-19 Vaccine Receipt by Health Care Personnel at a Major Academic Hospital During the First Months of Vaccine Availability
Source: JAMA Netw Open. 2021 Dec 1;4(12):e2136582. doi: 10.1001/jamanetworkopen.2021.36582 (PMC8637254; doi:10.1001/jamanetworkopen.2021.36582)
Supplement: Supplement. — eTable. Employee Prioritization Sequence Grid for Time to Receive COVID-19 Vaccine [file jamanetwopen-e2136582-s001.pdf]

Supplemental Online Content

Green-McKenzie J, Shofer FS, Momplaisir F, et al. Factors associated with COVID-19 vaccine receipt by health care personnel at a major academic hospital during the first months of vaccine availability. *JAMA Netw Open.* 2021;4(12):e2136582. doi:10.1001/jamanetworkopen.2021.36582

eTable. Employee Prioritization Sequence Grid for Time to Receive COVID-19 Vaccine

This supplemental material has been provided by the authors to give readers additional information about their work.

© 2021 Green-McKenzie J et al. *JAMA Network Open.*

eTable: Employee Prioritization Sequence Grid for Time to Receive COVID-19 Vaccine

Group ID	Targeted HCP Teams, Job titles, Departments, Groups	
Green Group	• Emergency Department (ED) staff<br>• Labor &Delivery (L&D) department staff; includes neonatologists working in delivery rooms<br>• Trauma Team (those that respond to traumas in ED e.g. neuro trauma, ortho trauma)	• COVID Testing Site personnel<br>• Lung Rescue Team, including Penn Star pilots<br>• Donor Retrieval Team<br>• Included housestaff in ED<br>• Does not include providers consulting in ED
Yellow Group	• Dedicated COVID floor and ICU unit staff: Nurses, Certified Nursing Assistants (CNA), Environmental Services<br>• COVID care teams: Advanced Practice Providers (APP) and physicians, including housestaff<br>• Respiratory Therapists (All)<br>• Anesthesia (physicians and APPs)<br>• Community health workers who perform in-home care or visits (Nurses, CNAs)<br>• OMFS office providers and clinical staff<br>• ENT office providers and clinical staff<br>• Endoscopy suite staff	• Endoscopists (GI, Pulmonary providers performing upper endoscopies)<br>• Stroke teams that respond in ED; Heart Rescue Teams<br>• Dental school clinical faculty, residents/trainees, and staff<br>• Corporate safety team (aka EHS) who perform fit testing<br>• Monoclonal Antibody Infusion suite staff (in PCAM)<br>• Housestaff from Oral Maxillofacial Medicine (OMFS), Otorhinolaryngology (ENT)<br>• Housestaff from Medicine & Family Medicine with in inpatient<br>• COVID care teams<br>• Anyone from Green group who did not get scheduled
Pink Group	• Providers and teams working with immunocompromised patient populations (neonates, oncology, transplant)<br>• Remainder of hospital floor and unit staff across entity<br>• All OR staff<br>• Remainder of the Security staff<br>• Remainder of the Front desk staff, red coats, greeters<br>• Remainder of EVS workers<br>• Pharmacy staff (outside those who work on floor/units)<br>• Food Services<br>• Laboratory personnel including Pathology and Lab Medicine<br>• All housestaff associated with programs and specialties in this group	• Cardiology (Echo, Stress, Noninvasive, Elextrophysiology, Catheterization laboratory), Interventional Radiology, Radiology<br>• Physical Therapy / Occupational Therapy, Case Management/Social Work/Patient and Guest Services/Pastoral Services<br>• Occupation Medicine/Employee Health department; Infection Control Department<br>• Remainder of Physician staff not already covered<br>• Ambulatory Practice Staff from Dickens, DGIM, Family Medicine,<br>• Maternal and Fetal Medicine, Obstetrics<br>• Administrators, Human Resources Department, Materials<br>• Management, CPD, Biomed, Information Services, Telecom (those located at entities)<br>• Facilities/physical plant staff<br>• Anyone from Yellow Group or Green Group who did not get scheduled
Blue Group	• Ambulatory Practice staff	• Medical students working primarily on clinical rotations
Gray Group	Corporate and all other non-clinical employees	

© 2021 Green-McKenzie J et al. *JAMA Network Open.*
